# Hydrogel-Coated Clips Are Associated with a Higher Risk of Dislocation After Ultrasound-Guided Breast Biopsy

**DOI:** 10.3390/diagnostics16121915

**Published:** 2026-06-20

**Authors:** Michael Swoboda, Johannes Deeg, Mark Panczel, Birgit Amort, Silke Haushammer, Valentin Ladenhauf, Malik Galijasevic, Pietro G. Lacaita, Daniel Egle, Afschin Soleiman, Michaela Kluckner, Leonhard Gruber

**Affiliations:** 1 Department of Radiology, Medical University Innsbruck, Anichstraße 35, 6020 Innsbruck, Austria; michael.swoboda@i-med.ac.at (M.S.);; 2 Department of Obstetrics and Gynaecology, Medical University Innsbruck, Anichstraße 35, 6020 Innsbruck, Austria; 3 Institute for Pathology, INNPath, University Hospital Tirol Kliniken, Anichstraße 35, 6020 Innsbruck, Austria; 4 Department of Vascular Surgery, Medical University Innsbruck, Anichstraße 35, 6020 Innsbruck, Austria

**Keywords:** breast biopsy, breast cancer, clip-marker, dislocation, migration

## Abstract

**Background**: Breast clip marker movement after ultrasound-guided biopsy can negatively affect lesion re-localisation rates and surgical outcomes, underscoring the need for improved understanding of the factors influencing clip displacement. Thus, this study aimed to compare four different breast clip markers and identify risk factors for clip migration and dislocation after ultrasound-guided placement. **Methods**: This retrospective study included 350 patients who underwent ultrasound-guided biopsy of a newly diagnosed breast lesion with placement of one of four types of breast clips (UltraClip Dual Trigger Biodur 108 Coil Marker [UC], TUMARK Professional [TP], TUMARK Vision [TV] and HydroMARK Breast Biopsy Site Marker [HM]). Clip migration and dislocation were assessed immediately after placement and during follow-up imaging for at least 3 months. A binary logistic regression analysis was performed to identify predictors of clip dislocation including lesional, perilesional and procedural parameters. **Results**: Clip migration rates were 26.0%, 18.0%, 10.0% and 25.0% and clip dislocation rates were 14.0%, 20.0%, 9.0% and 38.0% for UC, TP, TV and HM, respectively. Features significantly associated with clip dislocation included predominantly fatty surrounding tissue (*p* = 0.046) with low perilesional shear wave velocities (*p* = 0.054), smooth lesion contours (*p* = 0.041), soft lesion strain elastography (*p* =0.001), low clip-to-lesion-surface distance (*p* = 0.002) and the use of an HM breast clip (*p* = 0.032). **Conclusions**: The type of breast clip-marker, as well as perilesional and lesional characteristics, influence the likelihood of clip dislocation. Notably, the hydrogel-coated clip (HM) exhibited the highest rate of dislocation.

## 1. Introduction

Breast cancer is a significant public health problem worldwide, with a high incidence rate and mortality rate. Early detection and treatment of breast cancer are essential for improving patient outcomes, as surgical treatment is the cornerstone of curative treatment in non-metastasized breast cancer [1]. Complete tumour removal is critical to minimize the risk of disease recurrence and improve patient survival and surgical outcomes [2]. However, identifying the precise location of the tumour during surgery can be challenging, especially in cases where the tumour is small or non-palpable.

Since metal biopsy clips were first introduced in the late 1990s, various designs have been developed to address this challenge and enhance the reproducibility and reliability of marking breast cancer lesions [3]. Accurate and dependable placement of clips in tumours is crucial for guiding subsequent treatment and ultimately influencing patient prognosis [4,5]. Marking a malignant breast tumour or lymph node before neoadjuvant chemotherapy enables monitoring of treatment response and ensures accurate localisation, even in cases of complete imaging response where no residual malignancy is detected on imaging [6]. The clip markers help to accurately identify the location of the breast lesion during surgery, enabling complete tumour removal while minimising the removal of healthy breast tissue [7,8].

However, the use of clip markers is not without potential complications: one of the most significant complications associated with clip markers is clip dislocation or migration, which occurs when the marker moves from its original position in the breast tissue [9,10]. As a result, a reliable localisation of breast cancer is no longer ensured. The risk of clip displacement can be affected by various factors, including the type of clip marker used, the technique of placement, and patient-related factors such as breast size and tissue density [9,11].

While clip dislocations are rare [12,13], their potential impact on surgical outcomes and patient care underscores the need for greater understanding of this complication and strategies to minimize its occurrence. Therefore, the aim of this retrospective study is to compare four different breast clip markers and analyse the risk factors associated with dislocation and migration after ultrasound-guided placement.

## 2. Materials and Methods

### 2.1. Study Design

The study adhered to the principles of the Declaration of Helsinki (2013 revision) and received approval from the local Ethical Review Board on 13 September 2023 (ERB proposal 1176/2023). This retrospective, single-centre study included 350 patients who underwent ultrasound-guided biopsy of an unknown breast lesion with clip placement from January 2022 to May 2023.

### 2.2. Inclusion and Exclusion Criteria

All patients having undergone an ultrasound-guided biopsy with clip marker placement of an unknown breast lesion from January 2022 to May 2023 were retrospectively screened (*n* = 639). The initial target was to include 100 consecutive cases for each clip marker type. Since clip marker TUMARK Professional (TP) had been used in clinical routine significantly less than the others, only 50 cases were available and included. Additional clip types were available at our institution, but were used only sporadically. These were therefore excluded to avoid underpowered subgroup analyses. Patients who received neoadjuvant chemotherapy were excluded because treatment-related tissue changes (e.g., fibrosis, shrinkage) could confound the evaluation of clip displacement, which was not the focus of this study. Table 1 provides an overview of the inclusion and exclusion criteria.

### 2.3. Ultrasound Examination

Following international guidelines [14], ultrasound is performed in patients referred to our second-level centre for (1) routine or screening mammography with breast density ACR (American College of Radiology) C or D, (2) in case of a suspicious mammographic finding regardless of breast density, (3) referral due to a suspicious finding during gynaecological outpatient examination or (4) suspicious imaging findings from an extramural radiology practice. Ultrasound machines used were the Siemens Acuson S2000 or S3000 Evolution scanners (Siemens Healthineers International AG, Zurich, Switzerland) with a 18-6, 13.5 and 10 MHz high-resolution probe. When feasible, the highest-resolution probe is used. Systematic scanning is performed in an overlapping vertical and horizontal fashion in supine position. Breast lesions are documented in B-mode ultrasound in two planes, Doppler-sonography and elastography. Strain elastography (SE) was performed using light repetitive compression. Shear Wave Elastography (SWE) measurements were taken with a fixed 2–4 mm ROI placed centrally and perilesionally. The precise location of the lesions within the breast and breast tissue composition was documented.

### 2.4. Biopsy Procedure and Follow-Up Examinations

Breast biopsies with clip marker placement were carried out following international guidelines [15] and performed by radiologists specialized in breast imaging: L.G., with 8 years of breast-imaging experience; A.B., with 16 years; and S.H., with 11 years. After local anaesthesia, a small skin incision is made, and a 12 G or 14 G core-needle biopsy system (HistoCore Automatic Biopsy System, BIP GmbH, Türkenfeld, Germany) is used to acquire tissue specimens under constant ultrasound visualisation. A median of 5 core samples was obtained per lesion. The number of samples depended on lesion size and imaging suspicion. At our institution, clip placement after core-needle biopsy (CNB) is performed as part of standard clinical practice, as subsequent treatment planning is determined later by the interdisciplinary tumour board. After CNB, tumours are marked by placing one of four clips into the center of the lesion, if feasible:

- UltraClip Dual Trigger Biodur 108 Coil Marker (UC), Becton Dickinson, Franklin Lakes, NJ, USA;
- TUMARK Professional (TP), Somatex Medical Technologies, Berlin, Germany;
- TUMARK Vision (TV), Somatex Medical Technologies, Berlin, Germany;
- HydroMARK Breast Biopsy Site Marker (HM), Mammatome, Quickborn, Germany.

After undergoing CNB, all patients immediately receive mammography in lateromedial and craniocaudal views on a Mammomat Inspiration (Siemens Healthineers International AG, Zurich, Switzerland) or Seno Essential (General Electric Healthcare, Waukesha, WI, USA). Potential dislocation or migration of the clip was analysed during this initial imaging and subsequently at follow-up examinations at least 3 months apart from the initial placement. Clip movement within the lesion was defined as migration, whereas movement outside the lesion boundaries was defined as dislocation. If a clip was not placed initially within the lesion despite correct targeting, it was classified as a primary dislocation. If a clip, after being placed intralesionally, was no longer located within the lesion boundaries, it was considered a secondary dislocation. For multiple follow-up checks using different imaging modalities (e.g., ultrasound, confirmation mammography, CT, and/or MRI), the same modalities were compared, wherever possible.

### 2.5. Histopathologic Evaluation

Histopathologic analysis is performed by an experienced pathologist (A.S.) with gynaecologic specialisation and follows international guidelines and includes HE-staining and Elston and Ellis grading, if applicable. Immunohistochemical results of the index tumour, including oestrogen receptor, progesterone receptor, HER-2, and Ki-67 status, are routinely evaluated.

### 2.6. Statistical Analysis

Statistical analysis was carried out using GraphPad Prism 10.4.1 (GraphPad Software, Boston, MA, USA) and SPSS 27.0 (IBM Corporation, New York, NY, USA). Descriptive statistics were performed after classification of cases. Group characteristics were compared using an ordinary one-way ANOVA with a Holm–Sidak correction or Kruskal–Wallis test with Dunn’s post-test (in case of non-Gaussian distribution). Univariate analyses of binary and nominal variables were performed using cross-tabulations. A binary logistic regression was carried out to identify predictors of clip dislocation. Correction for multiple testing was performed where applicable. *p*-values < 0.05 are considered statistically significant, and *p*-values < 0.1 show a trend towards significance. Odds ratios (OR) with 95% confidence intervals (CI) were reported.

## 3. Results

### 3.1. Patient and Lesion Demographics

**Figure 1:**
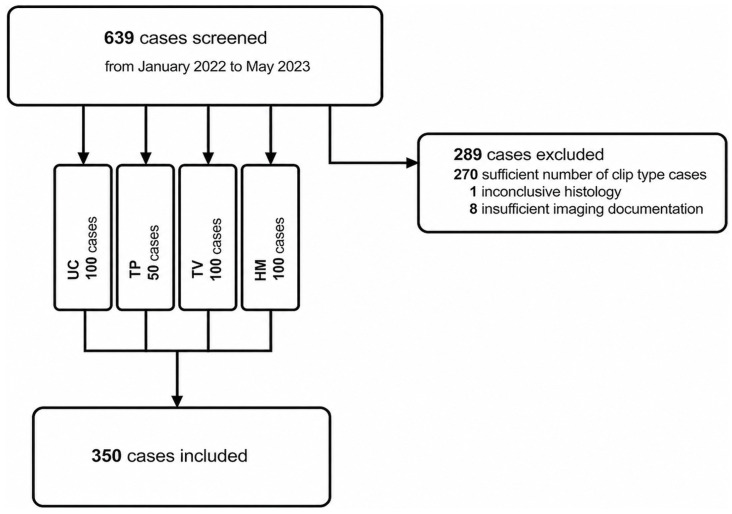
Flowchart of participant screening, exclusion and inclusion. UC: UltraClip Dual Trigger Biodur, TP: TUMARK Professional, TV: TUMARK Vision, HM: HydroMARK Breast Biopsy Site Marker.

Overall, 346 female (98.9%) and 4 male (1.1%) patients who had undergone biopsy and clip-marker placement due to a newly diagnosed breast lesion were included. Figure 1 provides an overview of the flowchart from initial retrieval to the final study cohort.

The average patient’s age was 59.9 ± 16.0 years (range 23 to 94 years). Lesions had an average size of 16.5 ± 10.8 mm (range 2 to 70 mm), an average volume of 2.2 ± 4.9 cm^3^ (range 0.1 to 47 cm^3^) and were more commonly located on the left side (n = 187 [53.4%]) and in the upper outer quadrant (n = 196 [56.0%]). The most common breast density was ACR C (35.3%) followed by ACR B (25.7%). Perilesional breast density was mostly predominantly fatty tissue (54%), followed by mixed (35.1%) and glandular tissue (10.9%). The most common histopathological diagnoses were malignant tumours (65.7%), with carcinoma of no special type (NST) being the predominant subtype (79.6%). For a detailed summary of patient and lesion characteristics and histopathological results, please refer to Table 2 and Table 3.

### 3.2. Dislocation/Migration Rates and Time Courses

After ultrasound-guided clip placement, clip migration was observed in 60 cases (17.1%), primary clip dislocation in 23 cases (6.6%), and secondary clip dislocation in 71 cases (20.3%). When comparing the four clip types, differences regarding migration and dislocation were noticed. While UC showed the highest rates of migration (26.0%), HM dislocated most frequently (38.0%). The mean distance of clip displacement was 4.2 ± 4.1 mm with a range of 0 to 20 mm. An overview of migration and dislocation rates is provided in Table 4.

**Figure 2:**
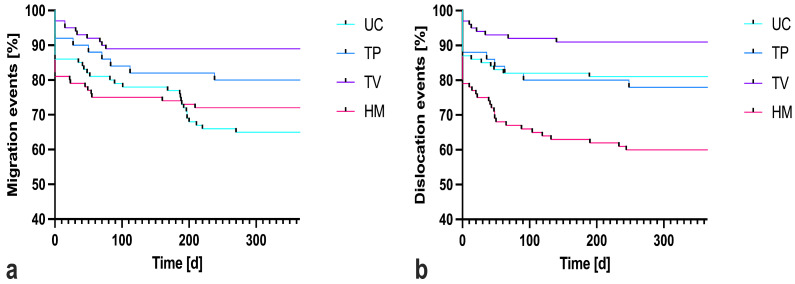
Kaplan–Meier curve illustrating the time course of clip migration (**a**) and clip dislocation (**b**) in four different clip types. UC: UltraClip Dual Trigger Biodur, TP: TUMARK Professional, TV: TUMARK Vision, HM: HydroMARK Breast Biopsy Site Marker.

There was a time dependency concerning clip migration and dislocation rates. A pronounced decline in event-free probability was observed during the early follow-up period, indicating that most migration and dislocation events occurred shortly after clip placement. Thereafter, the Kaplan–Meier curves reached a plateau, suggesting a substantially lower risk of late events. The overall median time to detection of migration was 22.5 days (range 0 to 538 days), with a median of 0 days for dislocation, including extra-lesional placement (range 0 to 248 days). For time to migration, TV demonstrated the highest probability of remaining free from migration over the entire follow-up period, followed by TP. In contrast, UC and HM showed a more pronounced decline in migration-free probability, indicating a higher rate of migration events. Similarly, in the analysis of time to dislocation, TV maintained the highest dislocation-free probability, while HM showed an increased risk of dislocation. UC and TP showed intermediate outcomes with relatively stable curves. Figure 2 illustrates the development over time.

### 3.3. Analysis of Cofactors of Clip Dislocation

Features that were associated with a clip dislocation were predominantly fatty perilesional tissue (*p* = 0.046), smooth lesion contour (*p* = 0.041), soft lesion SE (*p* = 0.001), lower perilesional SWE speeds (*p* = 0.054), clip HM (*p* = 0.032) and clip placement close to the lesion surface (*p* = 0.002). The results from a binary logistic regression analysis including lesion, perilesional and biopsy-related parameters can be found in Table 5.

## 4. Discussion

Numerous types of breast markers have been developed and are commonly utilized before neoadjuvant chemotherapy or surgical excision to mark the location of the targeted lesion [16,17]. In this study, we examined in 350 patients four different types of clip markers commonly used in the preoperative localisation of breast carcinoma and compared their migration and dislocation rates. Our findings indicate that clip dislocation was mainly found in the HydroMARK Breast Biopsy Site Marker (HM), with a dislocation rate of 38%, while the other three types of clips had much lower dislocation rates ranging from 9% to 20%. Regarding clip migration, the UltraClip Dual Trigger Biodur 108 Coil Marker (UC) showed the highest intralesional movement of the clip with a migration rate of 26%.

**Figure 3:**
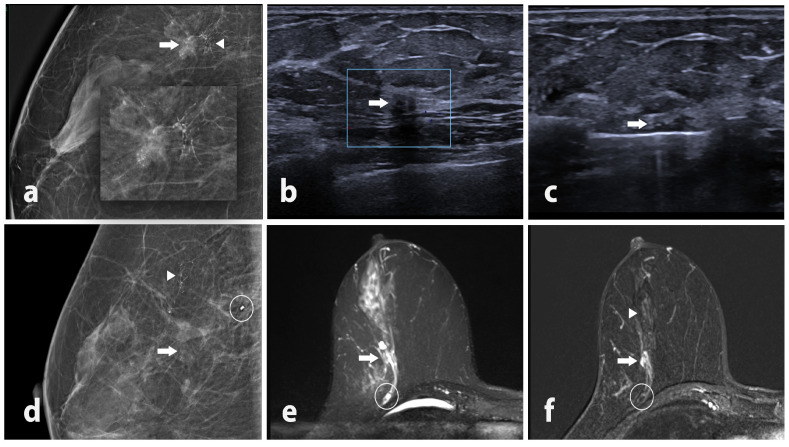
Lateral magnification mammography of the right breast (**a**) in a 53-year-old patient reveals a spiculated mass at the 9 o’clock position (arrow) with adjacent fine-linear branching calcifications (triangle). B-mode Ultrasound (**b**) identifies a hypoechoic lesion with 6 × 4 mm (arrow), leading to a semi-automatic ultrasound-guided biopsy (**c**) with intralesional placement of a HydroMARK Breast Biopsy Site Marker. Histopathological analysis confirmed an invasive carcinoma of non-specific type. Immediate lateromedial post-biopsy mammography (**d**) shows the mass (arrow) and fine-linear calcifications (triangle) but reveals displacement of the breast clip to a prepectoral location (circle). Breast MRI performed six days later for loco-regional staging, including TIRM axial (**e**) and post-contrast T1 subtraction (**f**) of the right breast, confirms the prepectoral displacement of the clip (circle) 23 mm relative to the residual lesion (arrow) and the presence of segmental non-mass Enhancement (triangle).

HM has been associated with a higher risk of clip dislocation [18], likely due to its unique design. It is made of a synthetic polyethylene glycol-based hydrogel with a titanium or stainless-steel coil embedded in its centre [19]. Upon placement at the biopsy site, the marker coat absorbs water from the surrounding tissue, causing it to expand within the biopsy cavity, which may cause the clip to migrate away from the initial insertion site along the biopsy canal [18]. This design has some advantages, such as improved visibility on imaging studies and better tissue marking [20]. However, the risk of clip dislocation must be carefully considered when selecting this clip marker and we showed that there is a significantly higher risk of clip dislocation (*p* = 0.032) with a dislocation rate of 38% when using HM. For an illustrative case, please refer to Figure 3.

Several studies have been performed to evaluate the frequency of clip migration and dislocation and the factors potentially associated with it [21,22]. Lee et al. showed that, in a meta-analysis of 3347 breast biopsy clips that were compared with dense breast, patients with fatty breast had a significantly higher odds of clip dislocation (*p* < 0.00001). This indicates that ACR A breasts may be a risk factor for clip dislocation [9]. In our study, we showed similar results, with perilesional predominantly fatty breast tissue leading to significantly higher clip dislocation rates (*p* = 0.046). When comparing both studies, caution is warranted, as Lee et al. use the term migration to describe clip movement away from the lesion. In the discussion, the original term used by Lee et al. was changed to dislocation, as this corresponds to the terminology used in our study. In our study, migration denotes intralesional movement of the clip, whereas dislocation refers to movement of the clip from the lesion. This observation may reflect the higher fibroglandular composition of dense breast tissue, which provides a more rigid and supportive environment for maintaining the position of the clip. In contrast, fatty breast tissue is softer and less structured, offering less resistance to movement or external forces [23].

Another significant factor identified in our study contributing to clip dislocation is a shorter distance between the clip and the lesion’s surface (*p* = 0.002). This finding is reasonable, as a smaller lesion increases the likelihood of the clip migrating to the surface, making it more prone to becoming perilesional. A study by Pinkney et al. [12], which reported a comparable average lesion size of 16.6 mm, found that lesion size was not a contributing factor to clip dislocation (*p* = 0.585). Similarly, our findings indicate that lesion volume is not a significant cofactor in clip dislocation (*p* = 0.131).

When analysing lesion characteristics, we found that imaging features typically associated with benign lesions [24], such as a smooth contour (*p* = 0.041) and soft lesion strain elastography (*p* = 0.001), as well as lower perilesional SWE speeds (*p* = 0.054), were associated with clip dislocation. Unlike malignant lesions, benign lesions lack invasive growth patterns and associated tissue reactions, such as desmoplastic changes or fibrosis [25].

Dense and fibrotic tissue may help to secure the clip, while benign lesions are typically softer and surrounded by fatty tissue, offering less resistance to clip dislocation [26]. Interestingly, our study demonstrated a significant association between increased stiffness on strain elastography and clip dislocation, whereas SWE values were not related to clip dislocation. This discrepancy could be explained by the fundamentally different physical principles underlying both techniques: Strain elastography reflects relative tissue deformation in response to external compression and is therefore highly sensitive to local mechanical factors. In contrast, SWE quantifies absolute shear-wave propagation speed, providing a more stable measure of intrinsic tissue properties and is less influenced by transient mechanical perturbations [27]. Furthermore, the compressibility of benign lesions [28] increases the likelihood of displacement when external forces, such as compression during post-biopsy imaging, are applied.

Clip displacement can occur within minutes of placement or be delayed for weeks or months later. Immediate clip dislocation is related to a phenomenon called the accordion effect, where the clip moves along the needle’s insertion axis following the decompression of the breast [29]. Although this phenomenon is frequently described in the literature following stereotactic core-needle breast biopsy [30], it may also account for the findings in our study, where the overall median time to detect primary clip dislocation was 0 days, and this is likely attributed to the compression applied during post-biopsy mammography. After a biopsy, the breast tissue is often more pliable or disrupted [31], and the placement of the biopsy marker might not yet be firmly secured in the tissue. When mammography is performed, the compression used to obtain clear images can lead to a displacement of the marker along the path of least resistance, which often aligns with the needle insertion axis [12]. This displacement could mimic an “accordion-like” motion, potentially causing the marker to shift from its original position.

To reduce the risk of clip displacement, several potential solutions have been proposed. One approach may be to postpone the confirmation mammography to a later date after the swelling of the hydrogel coating has stabilized. This would allow for a more accurate localisation of the clip marker and reduce the risk of dislocation. It is also important to note that the use of clip markers is only one part of a larger preoperative localisation strategy for breast carcinoma. Other techniques, such as wire localisation or radiofrequency identification, may be used in combination with clip markers to improve localisation accuracy and reduce the risk of margin involvement [32]. In cases of clip dislocation, these techniques may support treatment planning by facilitating accurate identification of the lesion.

Despite the potential solutions proposed to reduce clip movement, it is important to acknowledge that no single method is foolproof, and there is always a risk of clip migration or dislocation. As such, it is essential to remain vigilant and monitor patients closely for any signs of clip displacement during the preoperative period and during surgery.

### Limitations

There are some limitations to this study, and certainly, the retrospective study design represents a significant limitation. As a single-center study, case numbers per clip type were limited. A multicenter dataset would allow more balanced clip distributions. Furthermore, since primarily suspicious lesions are biopsied for further evaluation, benign findings were relatively underrepresented due to the absence of corresponding histological results. Additionally, a bias toward better-marked lesions or more visible clips is likely: clips that may have migrated unexpectedly far from a lesion were neither captured via ultrasound nor included in the study, potentially leading to an underestimation of the extent of clip dislocation. Moreover, only a limited selection of commercially available clips was used, which may limit the generalizability of the results to other device manufacturers. Additionally, the determination of clip position across different imaging modalities is not always perfectly comparable.

## 5. Conclusions

In conclusion, clip migration and dislocation are recognized complications following breast biopsy, posing significant challenges for surgical planning and precision. Our findings highlight a strong association between the use of hydrogel-coated clips and an increased likelihood of dislocation. Additionally, patient-related factors, such as the presence of perilesional fatty breast tissue and benign lesion characteristics, including smooth contours or soft lesion strain on elastography, were identified as contributing cofactors. These insights underscore the need for careful consideration of both clip type and individual patient and lesion attributes to minimize the risk of clip migration and dislocation and optimize surgical outcomes. Future research should focus on refining clip design and deployment techniques to address these factors effectively.

## Figures and Tables

**Table 1:** Inclusion and exclusion criteria.

Inclusion Criteria	Exclusion Criteria
Ultrasound-guided biopsy for a newly diagnosed breast tumour with same-day clip confirmation mammogram	Patient age under 18 years
Sufficient ultrasound documentation, including B-mode and shear-wave elastography imaging	Prior history of ipsilateral breast cancer
Follow-up imaging, including post-biopsy mammogram and (a) at least one follow-up examination, including post-wire mammogram or (b) one follow-up examination at least 3 months apart from the initial placement.	Insufficient ultrasound documentation
Available histological diagnosis	Patients undergoing neoadjuvant Therapy
	Inconclusive histology

**Table 2:** Characteristics of the included 350 patients and breast lesions.

Variables	n (%)
Sex	
Female	346 (98.9)
Male	4 (1.1)
Age group	
18–39	39 (11.1)
40–59	141 (40.3)
≥60	170 (48.6)
Breast lesions localisation	
Left breast	187 (53.4)
Right breast	163 (46.6)
Upper outer quadrant	196 (56.0)
Lower outer quadrant	72 (20.6)
Upper inner quadrant	38 (10.9)
Lower inner quadrant	44 (12.5)
Overall breast density	
ACR A	61 (18.9)
ACR B	83 (25.7)
ACR C	114 (35.3)
ACR D	65 (20.1)
Perilesional breast density	
Mostly glandular tissue	38 (10.9)
Mixed glandular and fatty tissue	123 (35.1)
Predominantly fatty tissue	189 (54.0)
Maximal lesion diameter	[mm]
Mean ± SD	16.5 ± 10.8
Range	2.0 to 70.0
Lesion volume	[cm^3^]
Mean ± SD	2.2 ± 4.9
range	0.1 to 47.0

**Table 3:** Distribution of histopathological diagnoses (n = 350).

Histopathological Diagnosis	n (%)
Benign lesions	107 (30.6)
Fibrous-cystic mastopathy	60 (56.1)
Fibroadenoma	33 (30.8)
Focal mastitis/fatty necrosis	7 (6.5)
other	7 (6.5)
High-risk lesions	13 (3.7)
Papillomatous neoplasia	7 (53.8)
ADH/epithelial proliferation	4 (30.8)
other	2 (15.4)
Malignant lesions	230 (65.7)
Invasive carcinoma, no special type (NST)	183 (79.6)
Ductal carcinoma in-situ	20 (8.7)
Invasive lobular carcinoma	15 (6.5)
other	12 (5.2)

**Table 4:** Comparison of migration and dislocation rates among clip types.

Clip Type	Overall Clips (n)	Migration Rate (%)	Dislocation Rate (%)	Clip Displacement Distance [mm]
UC	100	26	14	4.7 ± 4.7 mm
TP	50	18	20	3.6 ± 3.6 mm
TV	100	10	9	4.9 ± 2.6 mm
HM	100	25	38	4.0 ± 4.3 mm

**Table 5:** Binary logistic regression for clip dislocation cofactors.

					OR (95% CI)	
Variables	b	SE	*p*-Value	OR	Lower Value	Upper Value
Predominantly fatty tissue perilesional	1.105	0.553	0.046	3.020	1.021	8.932
Distance Lesion—Skin [mm]	0.007	0.054	0.901	1.007	0.905	1.119
Distance Lesion—Pectoralis [mm]	0.053	0.069	0.435	1.055	0.922	1.207
Lesion volume [cm^3^]	0.215	0.142	0.131	1.240	0.938	1.638
Vascularity						
None *			0.177			
Sparse	0.922	1.065	0.386	2.515	0.312	20.267
Moderate	−1.553	1.157	0.179	0.212	0.022	2.042
Strong	−1.538	1.056	0.145	0.215	0.027	1.703
Contour						
Smooth *			0.041			
Lobulated	−4.335	1.721	0.012	0.013	0.000	0.382
Irregular	−4.648	2.006	0.021	0.010	0.000	0.489
Margin						
Circumscribed *			0.264			
Partially circumscribed	−0.867	1.346	0.519	0.420	0.030	5.875
Diffuse	0.977	1.448	0.500	2.656	0.155	45.384
Strain Elastography						
Soft *			0.001			
Hard	4.677	1.591	0.003	107.408	4.748	2429.799
Same as surrounding	−1.239	1.631	0.448	0.290	0.012	7.082
Shear wave (SWE) assessment						
Intralesional SWE [m/s]	−0.005	0.030	0.860	0.995	0.938	1.055
Perilesional SWE [m/s]	−0.501	0.260	0.054	0.606	0.364	1.008
Clip type						
UC *			0.128			
TP	2.385	1.687	0.158	10.854	0.398	296.232
TV	2.100	1.575	0.182	8.170	0.373	179.095
HM	3.285	1.531	0.032	26.719	1.329	537.001
Procedure related						
CNB samples [n]	0.145	0.487	0.766	1.156	0.445	3.006
Needle gauge	−0.263	0.624	0.674	0.769	0.226	2.613
Clip-to-lesion-surface	−0.581	0.185	0.002	0.559	0.389	0.803
Distance [mm]						
Perilesional hematoma (yes)	−0.816	0.922	0.376	0.442	0.073	2.694

* Reference category.

## Data Availability

Data will be available on request to authors.

## Abbreviations

The following abbreviations are used in this manuscript:

UC	UltraClip Dual Trigger Biodur 108 Coil Marker
TP	TUMARK Professional
TV	TUMARK Vision
HM	HydroMARK Breast Biopsy Site Marker
SWE	Shear Wave elastography
ACR	American College of Radiology
SE	Strain elastography
CNB	Core needle biopsy
